# Large and non-specific somatic disease burdens among ageing, long-term opioid maintenance treatment patients

**DOI:** 10.1186/s13011-020-00311-4

**Published:** 2020-11-16

**Authors:** David Medved, Thomas Clausen, Anne Bukten, Ronny Bjørnestad, Ashley Elizabeth Muller

**Affiliations:** 1 Norwegian Centre for Addiction Research, Institute for Clinical Medicine, University of Oslo, Bygg 45, Ullevål sykehus, Kirkeveien 166, 0450 Oslo, Norway; 2 Division of Mental Health and Addiction, Oslo University Hospital, Oslo, Norway; 3 proLAR, Søgne, Norway; 4 Division of Health Services, Norwegian Institute of Public Health, Oslo, Norway

**Keywords:** Opioid maintenance treatment, Opioids, Somatic disease burden, Ageing, Chronic disease, Mental distress

## Abstract

**Objectives:**

To describe and explore somatic disease burdens of ageing long-term patients in opioid maintenance treatment (OMT), a unique population emerging in countries offering OMT as a long-term treatment.

**Methods:**

We used data from the Norwegian Cohort of Patient in Opioid Maintenance Treatment and Other Drug Treatment Study (NorComt). 156 patients enrolled for at least three of the past five years provided data during structured interviews, including on chronic conditions, somatic treatment received, mental distress (SCL-25), and treatment satisfaction. A somatic disease burden was calculated from a list measuring the recent severity of 16 somatic complaints. A hierarchical multiple linear regression analysis identified correlates of somatic disease burden.

**Results:**

Over half of patients reported at least seven somatic complaints. Reported somatic disease burden was associated with higher mental distress, more chronic conditions, fewer years in OMT, and treatment dissatisfaction. Age was unrelated, and there were few gender differences. These five variables explained 43.6% of the variance in disease burden.

**Conclusion:**

Long-term OMT patients experience a large range of somatic complaints, and at non-acute levels. As OMT secures longevity for opioid-dependent persons, the clinical focus must be adjusted from acute to chronic care. Providers must address how to optimize health and quality of life while in treatment, as treatment may last for many years.

**Supplementary Information:**

The online version contains supplementary material available at 10.1186/s13011-020-00311-4.

## Introduction

The general population is aging and so are opioid users [1]. Opioid use accounts for a significant amount of the global disease burden, and in 2016 there were 34 million opioid users worldwide [2]. In Norway, the gold standard treatment for opioid dependence, opioid maintenance treatment (OMT), is free and publicly provided, has no waiting lists, and is a life-long treatment for many. The mean age of OMT patients is therefore steadily increasing, while the intake of young patients is low, with nearly one third of patients now more than 50 years old [3]. The fact that the population of patients in OMT is ageing is a clear indicator of treatment stability and success [3, 4]. One recent qualitative study has reported that many long-term OMT patients in Norway attribute their survival to an older age to OMT [5].

In several other countries, patients entering OMT for the first time are also characterized by increasing age and more somatic comorbidities [6]; it follows that long-term patients will be those who have had their problems a long time [7]. Research has shown that mortality in this group of aging patients in OMT is more associated with comorbid somatic conditions, rather than ongoing illicit drug use [3, 8, 9]. A Norwegian comparison of 149 patients continuously in OMT with 51 “interrupters” showed a reduction in drug-related somatic problems for the continuous patients, but no difference in the amount of non-drug-related somatic problems between the two groups [10]. On one hand, this is a testament to OMT reducing illicit drug use over time. On the other hand, it speaks to the growing importance of recognizing OMT patients’ additional, non-drug-related health care needs – and likely screening for and treating them at an earlier age than what is necessary in non-OMT populations.

Some of the main somatic problems previously reported for this population in Norway have been hepatitis C, liver failure, cancer, kidney and lung disease [11, 12]. A recent Danish study reported an earlier onset of cardiovascular disease among hospitalized drug users compared to the general population [13], and it is likely that OMT patients also experience many other somatic problems at higher rates and at relatively young ages. Sexual dysfunction – a common side effect of OMT medications as well as associated with ageing – was also a common complaint among OMT patients according to a meta-analysis, although the authors noted that differences in sample ages prevented proper comparisons, and only men were included [14].

Research on the characteristics and needs of ageing and long-term OMT patients has been sparse, partly due to historically low survival rates. Another issue is that the lack of conceptual clarity hampers understanding and the measurement of long-term OMT patients’ needs [15]. First, no commonly accepted definitions of “long-term” exist; in some countries, “long-term” means several months, while in Norway, OMT is intended to be a life-long treatment. Second, “ageing”, “elderly”, and “older” are used interchangeably to describe patients, despite being defined as beginning from as young as 35 [16] to as old as 60. See Carew et al. [15] for a review of the numerous definitions of “old” used among this population.

With the OMT population aging, we need tailored healthcare that is adapted to their developing needs and characteristics [12, 17]. To begin planning such services, we first need more information directly from patients about their somatic health needs.

This paper has three aims pertaining to the somatic health challenge of long-term OMT patients, with data collected from patient interviews:

1. Identify and quantify chronic somatic conditions, health care utilization and treatment satisfaction among ageing OMT patients.
2. Explore participants’ overall, self-reported somatic disease burden.
3. Investigate factors associated with somatic disease burden.

## Methods

### Participants and setting

Cross-sectional data were drawn from the Norwegian Cohort of Patient in Opioid Maintenance Treatment and Other Drug Treatment Study (NorComt), a multi-center study involving 21 facilities across Norway providing OMT or residential treatment. NorComt methodology has been thoroughly described in earlier articles, and included three cohorts: patients entering OMT, patients entering residential drug treatment, and long-term OMT patients [18, 19]. This analysis is the first to report on the latter cohort of long-term OMT patients. As treatment interruptions are common in OMT – even among stable, long-term patients [20] – a previous drop-out was not an exclusion criterion. Rather, long-term patients were eligible if they were currently enrolled in OMT and had been enrolled for at least three of the past five years.

Data was collected through structured interviews utilizing a questionnaire. The questionnaire included numerous validated measures, self-developed measures, and measures commonly used in Norwegian health services. Previous publications of the larger NorComt project describe the questionnaire’s development thoroughly [18, 19]. The participating facilities were trained in questionnaire administration by the research team through a series of workshops and interview guides. Facility staff were instructed to invite all of their eligible long-term patients to participate. Participation rates were not consistently reported by the facilities, but the overall participation rate for the larger NorComt project (including the same facilities and staff) was 74%, and participation for this subgroup is likely similar. In total, 156 long-term patients currently in OMT were interviewed during 2012–2016.

### Measures

Somatic health problems and treatment involvement were self-reported through several checklists. The first list included ten chronic medical conditions relevant for ageing and/or substance-using populations, in which participants indicated whether they had the condition or if they did not know, and if they had received treatment for it in the past six months. Participants also self-reported if they had visited their general practitioner or another health care provider for somatic health issues in the past six months.

The second checklist included 16 somatic complaints common among chronic drug users, and including all major organ systems, based on the project leaders’ clinical experience. Participants indicated the extent to which they had been bothered in the past two weeks by each complaint. Answers were presented on a 0–4 Likert scale, with 0 corresponding to “not at all”, 1 “a little”, 2 “moderately”, 3 “a lot”, and 4 “very much”. A self-reported *somatic disease burden* variable was calculated as the sum of participants’ answers within this checklist, with a range of 0–64.

Three additional items captured participant evaluations of health status: “how satisfied are you with OMT in total?” (with three possible answers on a Likert-type scale), “how is your physical health now compared to before you entered OMT?” (three possible answers), and “how satisfied are you with your sexual functioning?” (five possible answers).

The interview questionnaire also included excerpts from the European version of the Addiction Severity Index [21] to collect substance use information. Participants reported their four most commonly used substances in the past six months from a list of 18 substances/categories. Each substance was presented with the amount of participants who reported it among their top four. Mental distress was measured by the Hopkins Symptoms Checklist-25 [22]. In the NorComt study we used a version of the SCL-25 with a composite score of 0–4 in which scores over 1.0 indicate clinically concerning mental distress [21, 23].

### Analysis

Descriptive statistics were used to report participants’ health variables. Subgroup analyses were conducted by gender, as we were particularly interested in any differences in types of somatic complaints, such as sexual dysfunction. To investigate factors associated with disease burden, a hierarchical multiple linear regression analysis was performed with somatic disease burden as the dependent variable. The first block was made with age as a forced entry variable. The rest of the variables were those with significant bivariate correlations and were requested with a stepwise entry. These variables were SCL25 score, amount of chronic conditions, total years in OMT, satisfaction with OMT treatment (reversed so that “dissatisfied” = 3 and “satisfied” = 0), gender, amphetamine use, and employment/studying. All statistics were performed with SPSS version 21.

## Results

There were 156 long-term OMT patients in our study, of which 59.6% were men (Table 1). The mean age in the group was 47.9 years (range: 31.6–64.3). The average length of time in OMT was 10.6 years, and ranged from 3 to 25 years. The majority were Nordic-born, single, and unemployed. Roughly one half (51.6%) had completed secondary education or higher. The patients were prescribed buprenorphine monopreparate, buprenorphine with naloxone, or methadone. The mean daily doses of buprenorphine monopreparate were 16.8 mg (*SD* 7.2), buprenorphine with naloxone 14.7 mg (*SD* 6.3) and methadone 100.5 mg (*SD* 37.5). The majority were prescribed methadone (57.7%). Patients were asked to name up to four substances they used most commonly during the past six months and more than half reported using cannabis. The second and third most commonly used substances were unprescribed and prescribed benzodiazepines; when combined, benzodiazepines were the most commonly used, by 60.6% of patients. Over 90% reported smoking cigarettes in the past six months. 60.5% of the patients had a SCL-25 score of 1.00 or more, indicating clinically concerning mental distress. Differences between genders were tested, but not found significant.

**Table 1:** Description of 156 long-term OMT patients in Norway (NorComt study, Norway, 2012-2016)

	Total N (%)	Women N (%)	Men N (%)
	156 (100)	63 (40.4)	93 (59.6)
Sociodemographic variables			
Age (mean, *SD*)	47.9 (*7.1*)	46.7 *(6.5)*	48.6 *(7.4)*
Nordic-born	150 (96.2)	62 (98.4)	88 (95.7)
Unmarried/without partner	133 (86.9)	55 (88.7)	78 (85.7)
Employed or studying	19 (12.2)	5 (8.1)	14 (15.4)
Secondary education or higher	80 (51.6)	28 (45.2)	52 (55.9)
Treatment variables			
Type of OMT medication			
Methadone	89 (57.0)	38 (60.3)	51 (54.8)
Buprenorphine monopreparate	42 (26.9)	16 (25.4)	26 (28.0)
Buprenorphine + naloxone	22 (14.1)	6 (9.5)	16 (17.2)
Other (Morphine)	1 (0.6)	1 (1.6)	0 (0.0)
Total years in OMT (mean, *SD*)	10.6 (*3.7*)	11.0 (*3.5*)	10.4 (*3.8*)
Health and substance use			
SCL25 score (mean, *SD*)	1.35 (*0.86*)	1.54 (*0.87*)	1.23 (*0.83*)
Substances used, past 6 months			
Cannabis	83 (53.2)	32 (50.8)	51 (54.8)
Any benzodiazepines	95 (60.6)	41 (65.1)	54 (58.1)
-- Unprescribed benzodiazepines	61 (39.1)	27 (42.9)	34 (36.6)
-- Prescribed benzodiazepines	44 (28.2)	22 (34.3)	22 (23.7)
Amphetamines	33 (21.2)	12 (19.0)	21 (22.6)
Alcohol	26 (16.7)	7 (11.1)	19 (20.4)
Heroin	26 (16.7)	10 (15.9)	16 (17.2)
Unprescribed OMT medicines	4 (2.6)	0 (0.0)	4 (4.3)
Cocaine	2 (1.3)	0 (0.0)	2 (2.2)
Ecstasy	2 (1.3)	0 (0.0)	1 (1.1)
Crack	0 (0.0)	0 (0.0)	0 (0.0)
LSD	0 (0.0)	0 (0.0)	0 (0.0)
Any nicotine use	147 (94.2)	60 (95.2)	87 (93.5)
Smoking	142 (91.0)	60 ( 95.2)	82 (88.2)
Smokeless tobacco	20 (12.9)	3 (4.8)	17 (18.3)

The majority of the 156 long-term opioid maintenance treatment patients interviewed were unemployed, had received less than a high school education, and exhibited clinically concerning mental distress. The average length of treatment was 10.6 years. Opioids and opiates were reported by only 19.3%

*OMT* opioid maintenance treatment

*SCL25* Hopkins Symptoms Checklist-25

### Chronic health conditions and health care utilization

Almost three quarters of the patients reported having at least one specified chronic condition (Table 2). The most commonly reported chronic condition was hepatitis C, reported by more than half (52.9%). However, very few of the patients with hepatitis C had received treatment for it in the past six months (12.2%). The second most common chronic condition was asthma (21.3%), with over 80% of the patients with asthma receiving treatment for it. Other common conditions were high blood pressure (10.3%), heart disease (5.8%), chronic obstructive pulmonary disease (COPD) (7.8%), and diabetes (2.6%), all conditions that commonly increase in prevalence with age. For all of these conditions more than half of the patients received treatment. Three patients reported having liver cirrhosis.

**Table 2:** Chronic conditions, health care utilization, and satisfaction (NorComt study, Norway, 2012-2016)

	N (%)	If yes, treatment past 6 mo. N (%)
Chronic conditions		
Any chronic condition	99 (73.5)	--
Amount of chronic conditions (mean, *SD*)	1.18 (*1.11*)	--
Hepatitis C	82 (52.9)	10 (12.2)
Unknown	8 (5.2)	--
Asthma	33 (21.3)	28 (84.8)
Unknown	8 (5.2)	--
Hepatitis B	22 (14.3)	--
Unknown	7 (4.5)	--
High blood pressure	16 (10.3)	9 (56.3)
Unknown	12 (7.7)	--
Chronic obstructive pulmonary disease	12 (7.8)	7 (58.3)
Unknown	8 (5.2)	--
Heart diseases	9 (5.8)	6 (66.7)
Unknown	8 (5.2)	--
Diabetes	4 (2.6)	3 (75.0)
Unknown	6 (3.9)	--
Liver cirrhosis	3 (1.9)	0 (0.0)
Unknown	12 (7.8)	--
HIV	3 (1.9)	2 (66.7)
Unknown	3 (1.9)	--
Cancer	1 (0.6)	0 (0.0)
Unknown	9 (5.8)	--
Health care utilization, past 6 months		
Appointment with general practitioner	126 (80.6)	
Other somatic health care appointment	82 (52.6)	
Satisfaction		
Overall satisfaction with OMT		
Satisfied	94 (61.8)	
Both satisfied and dissatisfied	46 (30.3)	
Dissatisfied	12 (7.9)	
Physical health compared to before OMT		
Better	95 (61.7)	
Same as before	18 (11.7)	
Worse	41 (26.6)	
Sexual functioning		
Very good	20 (14.2)	
Good	45 (31.9)	
Neither good nor poor	34 (24.1)	
Poor	19 (13.5)	
Very poor	23 (16.3)	

Almost three quarters of the patients reported having a chronic condition More than half reported having hepatitis C, for which only one eighth received treatment in the last six months. Most patients had seen a general practitioner in the past six months, and the majority was satisfied with OMT. A third of the patients reported having poor sexual functioning

*OMT* opioid maintenance treatment

80.6% of patients had seen a general practitioner in the past six months, and over half of them had other somatic health care appointments. 61.7% reported that their physical health was better compared to before they entered OMT; one tenth reported it was unchanged, and one quarter reported their physical health as having worsened. Over 60% of the patients also reported being satisfied with OMT treatment, about 30% were neutral, and only 7.9% were dissatisfied (Table 2).

When asked to evaluate their sexual functioning, almost one half (46.1%) of the patients reported good or very good sexual functioning, while almost one third (29.8%) of the patients reported poor or very poor sexual functioning. As shown in Supplementary Table 1, more men reported being satisfied with their sexual functioning than women (50 and 35% respectively).

The subgroup analyses by gender revealed few differences (Supplementary Table 1). More women reported having at least one chronic condition than men (82.5% of women compared to 66.7% of men). Women also reported having more chronic conditions (1.46 conditions on average compared to 0.99 for men). The only condition that was more prevalent among women was asthma (33.3% compared to 12.9% among men). There were no differences in the proportion of each gender receiving treatment for a particular condition.

### Overall self-reported disease burden

On a disease burden scale from 0 to 64, the average score was 13.6 (*SD* 9.3). Over half of the patients reported being bothered at least “a little” by at least seven somatic complaints in the past 6 months.

**Fig. 1:**
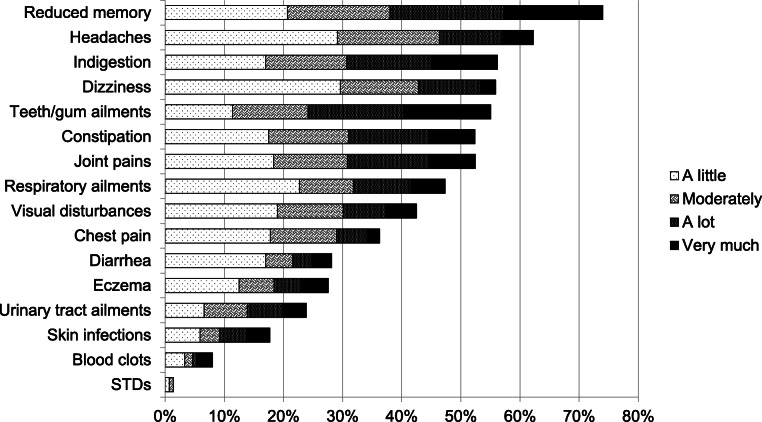
Self-reported disease burden. “How bothered are you by each of the following?” (NorComt study, Norway, 2012–2016). legend. Over 70% of patients reported being bothered by reduced memory, and over 50% reported being bothered by headaches, indigestion, dizziness, teeth and gum ailments, constipation and joint pain

Figure 1 displays each of the 16 somatic complaints, with “not at all” answers not shown. The most commonly reported complaint was reduced memory, with over 70% of the patients in OMT reporting being bothered at least “a little”. Other complaints reported by more than half of patients were headaches, indigestion, dizziness, teeth/gum ailments, constipation and joint pains. Between 30 and 50% of patients reported having visual disturbances, respiratory ailments and chest pains. 28% reported being bothered by eczema and 18% by skin infections; 32% reported being bothered by one or both. Almost 30% of the patients were bothered by diarrhea. Eight patients reported having blood clots.

### Factors associated with somatic disease burden

Five variables explained 43.6% of the variance in somatic disease burden among long-term patients in OMT (Table 3): the most explanatory variable was SCL25 score (β = 0.515, *p* < 0.001). This variable alone explained 33.3% of the variance. The second most important variable was number of chronic conditions (β = 0.288, p < 0.001). Total years in OMT was negatively associated to somatic disease burden (β = − 0.180, *p* = 0.011) and dissatisfaction with OMT positively associated (β = 0.149, p = 0,035). The regression equation was significant, F (5, 125) = 21.072, *p* < 0.001.

**Table 3:** Adjusted models explaining variance in somatic disease burden in long-term OMT patients in Norway^a^. ( NorComt study, Norway, 2012-2016)

	Model^b^				
	1	2	3	4	5
(Constant)	---	---	---	---	
Age	0.020	0.042	0.025	0.080	0.104
SCL25 score		0.586**	0.538**	0.562**	0.515**
Number of chronic condition			0.266**	0.278**	0.288**
Total years in OMT				-0.174*	-0.180*
Dissatisfaction with OMT					0.149*
Adjusted R^2^	0.000	33.3%	39.8%	42.0%	43.6%

Five variables explained 43.6% of the variance in somatic disease burden in long-term OMT patients. SCL25 score alone explained a third of the variance. Total years in OMT was a negative predictive factor

*p<0.05, **p<0.001

^a^*N*=131

^b^Significant bivariate variables not included in adjusted models: gender, amphetamine use, employment/studying

## Discussion

156 long-term OMT patients with an average of 10.6 years in OMT and an average age of 47.9 years reported a high prevalence of chronic somatic conditions. Three out of four had at least one chronic condition, with more than half reporting hepatitis C. Somatic disease burden analysis showed that rather than patients being highly plagued by a single somatic complaint, they were bothered by a wide range of problems on a non-acute basis. Mental distress had a strong relationship to experienced somatic disease burden.

Few studies have targeted long-term OMT patients, and those that have, have focused on the socioeconomic outcomes of treatment [24–26]. More common are studies about “elderly” patients, using a variety of cut-off and definitions of elderly and old [15]. Studies that compared older to younger patients have reported that older patients have a higher prevalence of mobility and sight problems [1], cardiovascular, gastrointestinal, and joint problems [27], and diabetes and liver disease [28]. Our study suggests that long-term OMT patients, even those younger than the ones in the previous studies, also suffer from a range of somatic problems. Sexual dysfunction, reported by 30% of our patients, may also be experienced by OMT patients at lower ages than non-OMT populations [29].

The self-reported disease burden represents a unique indicator of somatic complaints that long-term OMT users experience in their daily lives. The low score, but high amount of complaints, indicates that most patients were not highly plagued by one specific complaint or organ system. Rather than experiencing distinctly age-related problems or opioid-related problems, for example, their disease burdens were non-specific and varied on a group level. Low-level somatic complaints can accumulate and affect patients’ quality of life (perhaps more than serious single chronic condition which may be asymptomatic for years), and it is important for clinicians not to overlook these somatic complaints and to provide treatment and relief when appropriate. It is also important to keep in mind that some of these somatic complaints might be medication side effects.

Clinically concerning mental distress was strongly correlated to the experience of somatic disease burden. The association between anxiety, depression, and somatic complaints is well documented in the general population [30]. While psychiatric problems among OMT patients have been extensively reported [31–33], distress may also be heightened due to stigma, as recently reported in a similarly aged sample of Taiwanese OMT patients [34] and among others with substance use disorders [35]. Ageing patients in particular may experience stigma both from health care providers because they are drug users, and from other drug users because they are older or in OMT [11, 36, 37]. Such stigma may contribute to mistrust of and hesitation about using health care services [36], which in turn could explain the connection with a higher somatic disease burden. These relationships needs further investigating.

Patients had recently received treatment (56–84%) for most of their chronic conditions and the majority of the sample reported recent contact with some form of somatic health services. Given that this group of patients is in near constant contact with OMT services, we perhaps should be able to expect even higher rates of treatment for chronic conditions. At the same time, a six-month period might be too short of a window to capture those who need such treatment currently. Hepatitis C is a notable exception, and there is clearly potential for great improvement. Only one of every eight patients with hepatitis C had recently received treatment. One explanation for low uptake in our group might be that, due to an average age of only 49, they have not yet experienced the full complications of hepatitis C that can take decades to develop, such as liver cirrhosis, which was reported by less than 2%. Another explanation could be that the current gold standard, a 12-week treatment with direct-acting antiviral agents [38], was not yet available at the time of data collection. While all patients in OMT should be screened for hepatitis C, 5% of the patients in our sample still reported not knowing their hepatitis C status, even after at least three years in treatment.

Satisfaction rates with OMT were as high as those typically found in OMT patient satisfaction surveys [39], and predicted a lower somatic disease burden. High satisfaction is an important indicator of treatment outcome, but attention should also be paid to those who are dissatisfied, who may still have a higher risk of dropping out, even after many years in treatment. The experience of somatic complaints or medication side-effects may be a cause of treatment discontinuation, as reported in an early methadone study [40] and more recently among a prison sample [41]. This needs to be further explored, beginning by being continuously monitored by clinicians. User representatives can have important roles to play in aggregating patient feedback – perhaps especially feedback seen as negative, such as certain side-effects or dissatisfaction with treatment – as well as in helping clinicians decide how best to collect such feedback [42].

### Strengths and limitations

While a convenience sample, this sample shared many sociodemographic and treatment-related characteristics as the national OMT population [3] and as a peer-to-peer survey of 1011 patients in Norway conducted in the same time period [43]. This lends confidence to the generalization of our results to the long-term patient population as a whole, which is particularly encouraging given that participation rates were not reported by the interviewing facilities. Nevertheless, a cross-sectional design limits any firm claims of causality, and a confounder not captured by the questionnaire – such as years of drug use before enrolling into OMT – may better explain somatic disease burden than the variables in the regression model. Patients who agreed to participate may be more satisfied, and potentially less somatically burdened, than those who declined. Patients’ self-reports might have also been underestimates of chronic conditions. If true, the larger long-term OMT population may have an even higher somatic burden than observed. The relatively small sample size makes it difficult to identify significant associations between variables, and a larger size is needed in the future in order to explore subgroups such as gender. This was an exploratory analysis with many post hoc tests of association between the candidate predictor variables and somatic disease burden. As there was no control of Type I error rate, the chance of Type I error is inflated.

Finally, collecting patient-reported somatic complaints rather than clinical indicators is a novel technique that allowed us to construct a self-reported disease burden measure which provides information on the lived experiences of somatic problems. The main limitation to this technique is that the scale was locally developed and therefore the sample’s somatic disease burden cannot be directly compared to existing research. Future assessment of the scale’s measurement properties using data from the larger NorComt study is planned.

## Conclusion

Long-term OMT patients are a population that is likely experiencing more somatic health problems and at younger ages than the general populations, which brings new challenges to the treatment system as this population ages. In order to achieve further gains in survival and in quality of life, it is important that treatment providers address their somatic health, particularly access to hepatitis C treatment. Given the prevalence of chronic conditions, long-term patients should receive regular check-ups and screenings. The responsibility and capacity of somatic health services, OMT, and geriatric services to screen, refer, and treat such diseases must first be clearly defined. It is also important to focus not only on easily diagnosable diseases, but also on the range of complaints that many patients are used to living with, including side-effects from OMT medications and mental health. Doing so may encourage treatment retention and satisfaction in addition to promoting healthy longevity. Treatment services need to refocus the services to provide for patients with chronic conditions as well as over several decades of treatment, rather than services which are primarily geared towards acute conditions and shorter timeframes.

## Supplementary Information

**Additional file 1:** **Supplementary Table 1.** Chronic conditions, health care utilization, and satisfaction by gender (NorComt study, Norway, 2012–2016). legend. The subgroup analyses by gender revealed few differences in chronic conditions, treatment received, and satisfaction.

## Data Availability

Data is not yet available due to ongoing analysis.

## Abbreviations

NorComt: The Norwegian Cohort of Patient in Opioid Maintenance Treatment and Other Drug Treatment Study
OMT: Opioid maintenance treatment
SCL-25: The Hopkins Symptoms Checklist-25
COPD: chronic obstructive pulmonary disease
