# Supplementary material for: Large and non-specific somatic disease burdens among ageing, long-term opioid maintenance treatment patients
Source: Subst Abuse Treat Prev Policy. 2020 Nov 16;15:87. doi: 10.1186/s13011-020-00311-4 (PMC7667746; doi:10.1186/s13011-020-00311-4)
Supplement: Supplementary file 1 — Additional file 1: Supplementary Table 1. Chronic conditions, health care utilization, and satisfaction by gender (NorComt study, Norway, 2012–2016). legend. The subgroup analyses by gender revealed few differences in chronic conditions, treatment received, and satisfaction. [file 13011_2020_311_MOESM1_ESM.docx]

| **Supplementary table 1** Chronic conditions, health care utilization, and satisfaction by gender (NorComt study, Norway, 2012-2016) | | | | |
| --- | --- | --- | --- | --- |
|  | Women N (%) | If yes, treatment past 6 mo. N(%) | Men N (%) | If yes, treatment past 6 mo. N(%) |
|  |  |  |  |  |
| Chronic conditions |  |  |  |  |
| Any chronic condition* | 52 (82.5) | -- | 62 (66.7) | -- |
| Amount of chronic conditions (mean, SD)* | 1.46 (1.28) | -- | 0.99 (0.94) | -- |
| Hepatitis C | 35 (55.6) | 4 (11.4) | 47 (50.5) | 6 (12.8) |
| Unknown | 4 (6.3) | -- | 4 (4.3) | -- |
| Asthma** | 21 (33.3) | 16 (76.2) | 12 (12.9) | 12 (100.0) |
| Unknown | 2 (3.2) | -- | 7 (7.5) | -- |
| Hepatitis B | 10 (15.9) | -- | 12 (12.9) | -- |
| Unknown | 4 (6.3) | -- | 3 (3.2) | -- |
| High blood pressure | 8 (12.7) | 3 (37.5) | 8 (8.6) | 6 (75.0) |
| Unknown | 4 (6.3) | -- | 8 (8.6) | -- |
| Chronic obstructive pulmonary disease | 7 (11.1) | 4 (57.1) | 5 (5.4) | 3 (60.0) |
| Unknown | 3 (4.8) | -- | 5 (5.4) | -- |
| Heart diseases | 4 (6.3) | 2 (50.0) | 5 (5.4) | 4 (80.0) |
| Unknown | 2 (3.2) | -- | 6 (6.5) | -- |
| Diabetes | 3 (4.8) | 2 (66.7) | 1 (1.1) | 1 (50.0) |
| Unknown | 2 (3.2) | -- | 4 (4.3) | -- |
| Liver cirrhosis | 1 (1.6) | 0 (0.0) | 2 (2.2) | 0 (0.0) |
| Unknown | 4 (6.3) | -- | 8 (8.6) | -- |
| HIV | 2 (3.2) | 1 (50.0) | 1 (1.1) | 1 (100.0) |
| Unknown | 2 (3.2) | -- | 1 (1.1) | -- |
| Cancer | 1 (1.6) | 0 (0.0) | 0 (0.0) | 0 (0.0) |
| Unknown | 4 (6.3) | -- | 5 (5.4) | -- |
|  |  |  |  |  |
| Health care utilization, past 6 months | |  |  |  |
| Appointment with general practitioner | 53 (84.1) |  | 73 (78.5) |  |
| Other somatic health care appointment | 34 (54.0) |  | 48 (51.6) |  |
|  |  |  |  |  |
| Satisfaction |  |  |  |  |
| Overall satisfaction with OMT | |  |  |  |
| Satisfied | 32 (51.6) |  | 62 (68.9) |  |
| Both satisfied and dissatisfied | 23 (25.6) |  | 23 (25.6) |  |
| Dissatisfied | 7 (11.3) |  | 5 (5.6) |  |
| Physical health compared to before OMT | |  |  |  |
| Better | 42 (67.7) |  | 53 (57.6) |  |
| Same as before | 4 (6.5) |  | 14 (15.2) |  |
| Worse | 16 (25.8) |  | 25 (27.2) |  |
| Sexual functioning** |  |  |  |  |
| Very good | 11 (20.0) |  | 9 (9.7) |  |
| Good | 8 (14.5) |  | 37 (43.0) |  |
| Neither good nor poor | 14 (22.2) |  | 20 (23.3) |  |
| Poor | 9 (16.4) |  | 10 (11.6) |  |
| Very poor | 13 (23.6) |  | 10 (11.6) |  |
| OMT: opioid maintenance treatment. *p<0.05, **p<0.01, ***p<0.001. | | | | |

*Supplementary table 1 legend. The subgroup analyses by gender revealed few differences in chronic conditions, treatment received, and satisfaction.*
